# An Evaluation of the Benefits of Simultaneous Acquisition on PET/MR Coregistration in Head/Neck Imaging

**DOI:** 10.1155/2017/2634389

**Published:** 2017-07-18

**Authors:** Serena Monti, Carlo Cavaliere, Mario Covello, Emanuele Nicolai, Marco Salvatore, Marco Aiello

**Affiliations:** IRCCS SDN, Naples, Italy

## Abstract

Coregistration of multimodal diagnostic images is crucial for qualitative and quantitative multiparametric analysis. While retrospective coregistration is computationally intense and could be inaccurate, hybrid PET/MR scanners allow acquiring implicitly coregistered images. Aim of this study is to assess the performance of state-of-the-art coregistration methods applied to PET and MR acquired as single modalities, comparing the results with the implicitly coregistration of a hybrid PET/MR, in complex anatomical regions such as head/neck (HN). A dataset consisting of PET/CT and PET/MR subsequently acquired in twenty-three patients was considered: performance of rigid (RR) and deformable (DR) registration obtained by a commercial software and an open-source registration package was evaluated. Registration accuracy was qualitatively assessed in terms of visual alignment of anatomical structures and qualitatively measured by the Dice scores computed on segmented tumors in PET and MRI. The resulting scores highlighted that hybrid PET/MR showed higher registration accuracy than retrospectively coregistered images, because of an overall misalignment after RR, unrealistic deformations and volume variations after DR. DR revealed superior performance compared to RR due to complex nonrigid movements of HN district. Moreover, simultaneous PET/MR offers unique datasets serving as ground truth for the improvement and validation of coregistration algorithms, if acquired with PET/CT.

## 1. Introduction

Integration of multimodal information carried out from different diagnostic imaging techniques is essential for a comprehensive characterization of the region under examination. Therefore, image coregistration has become crucial both for qualitative visual assessment [1] and for quantitative multiparametric analysis in research applications [2, 3] and clinical diagnosis, staging, and follow-up. Coregistration of complex data, such as diagnostic images, is typically computationally intense, and its result could also be inaccurate. This problem is intrinsically overcome by hybrid systems that allow acquiring simultaneously images that share the same coordinate system [4, 5].

In this field, the recently introduced integrated PET/MRI scanners represent the new frontier of molecular imaging. This new technology allows achieving in one-shot both functional information provided by positron emission tomography (PET) imaging and morpho-functional information with excellent soft tissue contrast provided by magnetic resonance imaging (MRI), increasing patient's compliance. The advantages of such a technology go beyond the mere combination of functional and morphological imaging: considering the wide range of MRI sequences and PET radiotracers available [6], the functional information of both MRI and PET may complement each another; moreover, due to the high spatial and contrast resolution of MRI, PET/MR imaging is becoming a straightforward clinical indication for local staging in complex anatomical regions such as head/neck [7], where it can help in delineating the tumor extent and lymph node involvement from the surrounding tissue [8–11]. Furthermore, PET/MRI can be useful for radiation therapy and presurgical treatment planning in head and neck cancer patients [12, 13].

With respect to separate acquisition of PET and MR, hybrid systems can certainly overcome the computational problem of the PET and MR coregistration, carrying out at same time PET and MR images of the same anatomical district that are, therefore, ideally coregistered.

Despite the undeniable advantages of hybrid solutions, their cost effectiveness is still far to be proven and the coregistration of multimodal information is frequently retrospectively obtained via software, combining images from a PET scanner with preexisting CT and MR, thus reducing the cost for new technology purchasing while offering renewed opportunities to advance PET, especially in underserved areas or under increasing economic constraints [14]. The problem of multimodal coregistration via software is commonly approached by algorithms consisting of an affine or rigid transformation followed by a free form deformation and using mutual information [15–17] as similarity measure. These algorithms are based, when available, on the coregistration of the anatomical information of CT component of PET/CT with MR, while the PET component can be transformed with the resulting deformation field, in order to guarantee more accurate coregistration of PET/MR data [13]. Retrospective coregistration via software has shown good performances also in the HN district [13, 18], but it is particularly challenging and technically demanding, mainly because of the varied patient positions used for the various scanners and the anatomic complexity of this region [10], which is subject to respiration, swallowing, and intrinsically nonrigid movements [19].

Looking at this scenario, our study is aimed to assess the performance of the state-of-the-art coregistration methods between PET and MR acquired as single modalities, comparing them with the intrinsic coregistration carried out by a hybrid PET/MR system, which is assumed to represent a ground truth for the assessment of retrospective coregistration. In particular, the performance of the state-of-the art rigid and deformable registration algorithms, implemented by a commercial software and an open-source registration package, was evaluated to appreciate their clinical suitability. Our work is based on a dataset of PET/MR and PET/CT of the HN district acquired during the same session, in order to exploit just a single administration of FDG-PET radiotracer.

## 2. Materials and Methods

The study was approved by the Institutional Review Board: 23 patients, with histologically confirmed HN malignancy (in early staging and in follow-up), were studied, after obtaining written informed consent.  Table 1 shows clinical details for each patient.

### 2.1. Imaging Protocol

All subjects underwent a single-injection dual imaging protocol including PET/CT and subsequent PET/MR, so that no additional injection was required and any additional radiation exposure for the patients was avoided. The examination protocol consisted of the following steps: patients fasted for at least 6 h before scanning; just before the injection, the blood glucose level was measured in order to ensure a value below 150 mg/dL; and the patients were injected with about 400 MBq of [18F]-FDG, depending on their body weight. After an uptake period of 80 minutes, patients underwent PET/CT scanning and, soon after PET/CT, they underwent PET/MR examination.

#### 2.1.1. PET/CT Acquisition

PET/CT acquisition was performed on a Gemini TF (Philips Medical Systems, Best, The Netherlands). PET data was acquired in sinogram mode for 15 minutes with a matrix size of 144 × 144. A 3-dimensional attenuation-weighted ordered-subsets expectation maximization iterative reconstruction algorithm (AW OSEM 3D) was applied with 3 iterations and 21 subsets, Gaussian smoothing of 4 mm in FWHM, and a zoom of 1. The CT consisted of a low-dose scan (120 kV, 80 mA). Patient position was supine with his arms resting at the side.

#### 2.1.2. PET/MR Acquisition

PET/MR was performed on a Biograph mMR (Siemens Healthcare, Erlangen, Germany). Bed position was established in order to get a full coverage of the head/neck region. Also, these PET data were reconstructed with an AW OSEM 3D iterative reconstruction algorithm applied with 3 iterations and 21 subsets, Gaussian smoothing of 4 mm in full width at half maximum, and a zoom of 1. MR attenuation correction was performed via a segmentation approach based on 2-point Dixon MRI sequences. The MRI protocol was performed with dedicated head and neck coils. The MRI sequences taken into account for this study were T2-weighted short time inversion recovery (STIR) acquired in coronal direction (TR/TE/TI = 5000/84/220, one acquired signal, voxel size = 0.4 × 0.4 × 3.5 mm).

### 2.2. Data Processing

Image registration strategies were developed based on CT and MR data only, with the MR coronal STIR acquisition serving as fixed image and the CT as moving, whereas the PET component from PET/CT data was transformed only later by the resulting deformation field into the same coordinate system of PET/MR. The entire registration process was performed twice, using two different tools: the freely available, open-source registration package Elastix (http://elastix.isi.uu.nl) [20] and the tool for deformable image registration included in the commercial software XD3 (Mirada Medical Ltd., Oxford, United Kingdom) [21]. In the following, we will refer to the set composed by PET and MR acquired on the hybrid PET/MR scanner as PETMRo and to the set composed by PET from PET/CT retrospectively coregistered to MR from PET/MR as PETMRreg. Moreover, registration performed by means of Elastix or Mirada will be superscripted with ELX or MRD, respectively, while the suffixes RR and DR will indicate rigid and deformable registration, respectively.

#### 2.2.1. Image Registration with Elastix

Elastix is a command line-driven program based on the Insight Toolkit (ITK) registration framework (open source: National Library of Medicine, www.itk.org). The registration parameters were selected based on previous work on multimodal deformable image registration for integration of PET/MR into radiotherapy treatment planning for head and neck [13]. First, a rigid registration (RR) was performed and the resulting transform was used as a starting point for deformable registration (DR), by means of B-spline transform [15]. Both RR and DR were performed with a three-level multiresolution approach, using Gaussian smoothing (sigma = 8.0, 4.0, and 4.0 in *x* and *y* direction and sigma = 2.0, 1.0, and 0.5 in *z* direction to take into account voxel anisotropy) without downsampling. A localized version of mutual information (LMI) was considered as similarity measure (Mattes Mutual Information) [22], and a stochastic gradient descent optimizer [23] was chosen to minimize it. In detail, for RR, LMI metric was computed with 64 bins, 2000 samples, and a maximum of 500 iterations for each resolution. For DR, a bending energy penalty (BEP) term was calculated [15] to regularize the transformation; the metric (sum of LMI and BEP) was computed with 60 bins, 10,000 samples, and a maximum of 5000 iterations for each resolution. After the deformation field that maps the CT into the space coordinates of MR was computed, the PET from PET/CT was accordingly warped using Transformix, another command line-driven program based on ITK that applies a known transformation to an input image.

#### 2.2.2. Image Registration with XD3

XD3 is a Mirada's commercial platform that provides a full suite of practical applications for multimodal image viewing, including rigid and deformable registration. After a first step of RR between MR and CT, a DR was performed using Mirada's multimodal deformable image registration algorithm that optimizes a proprietary form of a mutual information-based similarity function [15, 16, 24] over a radial basis function (RBF) transformation model. Default parameters for MR-CT unsupervised registration were used. Finally, the PET from PET/CT was automatically warped, once the transformation that registers CT to MR was computed.

### 2.3. Image Evaluation

Registration accuracy was qualitatively and qualitatively evaluated in five sets of images for each patient: PETMRo, PETMRreg_ELX_^RR^, PETMRreg_ELX_^DR^, PETMRreg_MRD_^RR^, and PETMRreg_MRD_^DR^. Qualitative evaluation was performed by two clinical reviewers: one is a nuclear medicine physician who is also licentiate in diagnostic radiology and the other is a radiologist who is also licentiate in nuclear medicine. Images were analyzed in the coronal plane on the freely available medical imaging platform medInria [25], which allows visualization and fusion of both NIfTI files and DICOM files. The observers reviewed the five image set for each patient evaluating the alignment of the major anatomical structures. Then they identified, in PET and MR images, the localization and the extent of the primary tumor and metastasis to regional lymph nodes, and, on these bases, they independently rated the registration quality of each tested method using the scoring system defined in Table 2. Neither reader was aware of the results of other imaging studies, histopathologic findings, or clinical data.

In order to obtain also a quantitative evaluation of the registration accuracy, the two clinicians were asked to segment the primary tumor of each patient using the freely available software ITK-SNAP (http://www.itksnap.org) [26]. The radiologist manually contoured the lesion in the T2-weighted coronal image. The nuclear medicine physician used the user-guided 3D active contour segmentation implemented in ITK-SNAP to semiautomatically segment the primary tumor, after having initialized the process with the placement of a spherical seed. For each patient, he repeated five times the operation (once for each PET of the five sets). The obtained segmentations were then used to compute the Dice score (Dice, 1945 number 39) between MR and PET from each set.

### 2.4. Statistical Analysis

A Friedman statistics with successive multicomparative analysis were used to evaluate the statistical differences in visual ratings between implicitly coregistered PET/MR and the results of coregistration software. Further comparisons between the single steps of the different coregistration software were evaluated by means of a Wilcoxon's signed-rank statistic test, as done in [27]. Similarly, statistical differences in Dice scores were tested with ANOVA and paired Student's *t*-test. Statistical analysis was performed using Matlab (MATLAB R2014b, Math-Works, Natick, MA). Differences at *p* < 0.05 were considered to be statistically significant.

## 3. Results

Table 3 shows the mean qualitative scores over the two observers and the Dice score for each patient and for each set considered.

Comparing PETMRo with registrations performed by Elastix and Mirada, their differences resulted to be statistically significant both at qualitative (Friedman test *p* values: PETMRo/PETMRreg_ELX_^RR^/PETMRreg_ELX_^DR^ = 2.92 · 10^−7^, PETMRo/PETMRreg_MRD_^RR^/PETMRreg_MRD_^DR^ = 4.56 · 10^−7^) and quantitative analysis (ANOVA *p* values: PETMRo/PETMRreg_ELX_^RR^/PETMRreg_ELX_^DR^ = 4.83 · 10^−11^, PETMRo/PETMRreg_MRD_^RR^/PETMRreg_MRD_^DR^ = 3.80 · 10^−12^). For each patient, the scores highlighted that PETMRo set showed a higher (at the most equal) registration accuracy than the other fused sets of images.

This superiority was statistically significant in all the comparison for qualitative (Wilcoxon test *p* values: PETMRo/PETMRreg_ELX_^RR^ = 1.25 · 10^−7^, PETMRo/PETMRreg_ELX_^DR^ = 0.02, PETMRo/PETMRreg_MRD_^RR^ = 2.46 · 10^−6^, PETMRo/PETMRreg_MRD_^DR^ = 3.50 · 10^−5^) and quantitative scores (*t*-test *p* values: PETMRo/PETMRreg_ELX_^RR^ = 1.57 · 10^−10^, PETMRo/PETMRreg_ELX_^DR^ = 2.22 · 10^−8^, PETMRo/PETMRreg_MRD_^RR^ = 1.45 · 10^−10^, PETMRo/PETMRreg_MRD_^DR^ = 1.20 · 10^−9^).

The registration results of PET with MR images after a RR step showed an overall misalignment due to different patient positioning, both for Elastix and Mirada results, with differences between the two methods that were not statistically significant both at the qualitative (Wilcoxon test *p* value PETMRreg_ELX_^RR^/PETMRreg_MRD_^RR^ = 0.73) and at the quantitative scores (*t*-test *p* value PETMRreg_ELX_^RR^/PETMRreg_MRD_^RR^ = 0.75).

If a DR step was performed, a significant improvement could be obtained, but also unrealistic deformations or moderate and smooth volume expansions and compressions could occur, leading to a good or sufficient alignment of major anatomical structures but local misregistration of tumors. However, looking at the scores of the single patients, after a DR step, the accuracy of registration tended to improve for both Elastix and Mirada results. This improvement was statistically significant for registration performed with Elastix (Wilcoxon test *p* value PETMRreg_ELX_^RR^/PETMRreg_ELX_^DR^ = 0.01, *t*-test *p* value PETMRreg_ELX_^RR^/PETMRreg_ELX_^DR^ = 1.4 · 10^−3^) and not statistically significant for Mirada (*p* value PETMRreg_MRD_^RR^/PETMRreg_MRD_^DR^ = 0.85, *p* value PETMRreg_MRD_^RR^/PETMRreg_MRD_^DR^ = 0.09)

Comparing the results obtained from the two registration tools, the scores of the DR output with Elastix were generally higher than those of the DR outputs, but the arisen differences between these two sets were statistically significant only at the qualitative assessment (Wilcoxon test *p* value PETMRreg_ELX_^DR^/PETMRreg_MRD_^DR^ = 0.02, *t*-test *p* value PETMRreg_ELX_^DR^/PETMRreg_MRD_^DR^ = 0.32.

**Figure 1:**
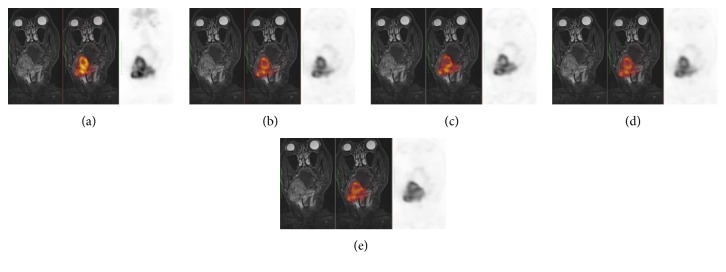
Example of qualitative well ranked coregistration results. From left to right: coronal MR image, fused PET/MR, and PET image from (a) PETMRo, (b) PETMRreg_ELX_^RR^, (c) PETMRreg_ELX_^DR^, (d) PETMRreg_MRD_^RR^, and (e) PETMRreg_MRD_^DR^. Both RR and DR with Elastix, (b) and (c), respectively, and Mirada, (d) and (e), respectively, show results comparable with intrinsic coregistration of simultaneous PET/MR (a). The Dice scores for this case are PETMRo = 0.95, PETMRreg_ELX_^RR^ = 0.85, PETMRreg_ELX_^DR^ = 0.86, PETMRreg_MRD_^RR^ = 0.89, and PETMRreg_MRD_^DR^ = 0.90.

**Figure 2:**
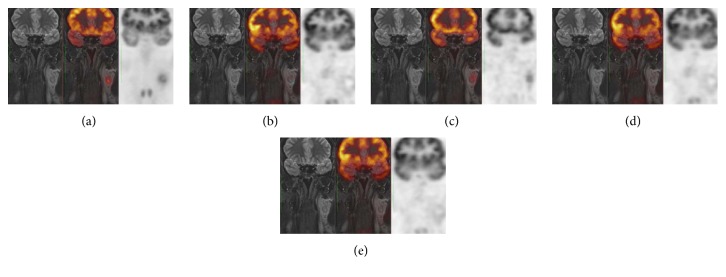
Example of poorly ranked coregistration results. From left to right: coronal MR image, fused PET/MR, and PET image from (a) PETMRo, (b) PETMRreg_ELX_^RR^, (c) PETMRreg_ELX_^DR^, (d) PETMRreg_MRD_^RR^, and (e) PETMRreg_MRD_^DR^. RR with Elastix (b) and Mirada (d) shows an overall misalignment of the brain contour between PET and MR. This misalignment is only partially recovered by DR with Mirada (e) and better recovered by DR with Elastix (c). However, the lymph node tumor is completely absent in the PET component of PETMRreg_MRD_^RR^ and its localization is not perfectly corresponding in PETMRreg_ELX_^DR^ (c) as in PETMRo (a). The Dice scores for this case are PETMRo = 0.79, PETMRreg_ELX_^RR^ = 0.26, PETMRreg_ELX_^DR^ = 0.52, PETMRreg_MRD_^RR^ = 0.19, and PETMRreg_MRD_^DR^ = 0.25.

In conclusion, while at the quantitative assessment, hybrid PET/MR definitely outperforms retrospective registration; at the qualitative score in the 25% of the cases, all retrospective coregistration methods showed results that were comparable with PETMRo in terms of alignment of major anatomical structures and tumors (Figure 1). In the remaining 75%, PETMRo exhibited an overall superiority. In detail, the 17% of these cases showed a slightly better performance of Elastix-based registration, in particular the DR step, in comparison with Mirada; one case showed a better performance of Mirada; in the remaining ones, problems of misalignment in RR steps and/or volume variations for DR steps were visible (Figure 2).

## 4. Discussion

In this work, four different strategies for the coregistration of PET and MR in the HN region were qualitatively and quantitatively evaluated, with the purpose of comparing them with the intrinsic coregistration of simultaneous PET/MR, which is assumed to represent a ground truth for the assessment of retrospective coregistration.

To our knowledge, this is the first reported study to have investigated the validity of retrospectively coregistered PET/MR of HN district using images obtained from different modalities in terms of localization and extent of the primary tumor and metastasis to regional lymph nodes and to have compared the accuracy of anatomical structure alignment and tumor localization with the intrinsic coregistered simultaneous PET/MR.

Kanda et al. [9] assessed the clinical value of retrospective image coregistration of neck MRI and [18F]-FDG PET for loco-regional extension and nodal staging of neck cancer in 30 patients, comparing it with PET/CT fusion. Although they used manual registration, they hypothesized that simultaneous PET/MR technology would minimize the drawbacks of retrospective PET/MR coregistration strategy, such as local misregistration, generating better-quality fusion images, as can be confirmed by our study.

The same has been studied by Loeffelbein et al. [11] that compared their retrospective coregistration results obtained by a commercial software with side-by-side analysis of single modality PET and MRI in a group of thirty patients.

In neither of these two studies, the authors had data sets coming from simultaneous PET/MR available to use as gold standard for the evaluation of registration performances.

Leibfarth et al. [13] developed an accurate and robust registration strategy on a dataset of eight patients consisting of an FDG PET/CT and a subsequently acquired PET/MR of HN with the aim of integrating combined PET/MR data into RT treatment planning. We started from this work for the implementation of registration with Elastix, but we took advantages of a wider dataset and we also evaluate registration performed by a commercial software optimized for a clinical workflow.

Our results showed that comparison of rigid versus deformable registration revealed superior performance for deformable registration both for Elastix and Mirada. This is due to the complex movements of this region, which are intrinsically nonrigid and hence cannot be completely recovered by a rigid transformation with only six degrees of freedom. With regard to deformable registration, although Elastix showed better performance than Mirada at least in the qualitative evaluation, it was computationally more intense. The tested software used different similarity measures and different transform basis: although Mirada uses RBF transformation model, which is more accurate and faster than the B-Spline used in this work for Elastix registration, it is completely embedded. Consequently, internal registration parameters, such as deformation field smoothness, degrees of freedom, and similarity function sensitivity, are automatically tuned by the software on the basis of the considered modalities. On the other side, the registration scheme and parameters used for DR in Elastix are the result of a previous optimization study [13] and are designed for the specific application in MR-CT registration of HN district; in particular, B-spline parametrization in conjunction with BEP is chosen to favor a smooth and reasonable transform. LMI is advantageous in the case of spatial intensity distortions and for multimodal registration if one intensity class corresponds to a specific tissue type in one imaging modality and to different tissue types in the other imaging modality [13]. Moreover, as expected, coregistered PET/MR images from hybrid scanner carried out the best performances, since they are inherently free from the problems of misalignment, local misregistration, and unrealistic deformation field.

We believe that performances of software-based coregistration method in districts subject to nonrigid movements, such as HN, could be undoubtedly improved by means of support structures, as head masks, designed for immobilizing the patient during the acquisitions. In addition, registration algorithms could benefit from users' supervision for preliminary manual step, in order to start from an optimal rigid alignment that could improve the performances of successive automatic deformable steps, making them more feasible in terms of computational time. Both these issues are out-of-the-scopes of this work that, although it is aimed to evaluate the clinical suitability of retrospective coregistration in comparison to intrinsic coregistration of simultaneous PET/MR, limits the investigation to a fully automated perspective.

In conclusion, our findings show that, regarding the complex case of PET/MR of HN district, there is a wide room for improvement of software-based coregistration algorithms, since, at present, they are definitely outperformed by the intrinsic coregistration of simultaneous PET/MR that overcomes the above-named problem of retrospective coregistration, as hypothesized in previous works [9, 10]. In this direction, simultaneous PET/MR imaging, which hence offers unique datasets when acquired together with PET/CT during the same session, could also serve as ground truth for the validation of improved coregistration algorithms.

## Figures and Tables

**Table 1:** Patient's cohort examined during this study. For each patient, in addition to personal details, the site of malignancy is specified.

ID	Age	Sex	Site
*pt1*	60	M	Rhinopharynx
*pt2*	68	M	Oropharynx
*pt3*	61	M	Tongue
*pt4*	70	M	Larynx
*pt5*	52	F	Hypopharynx
*pt6*	61	M	Larynx
*pt7*	56	M	Tongue
*pt8*	35	F	Larynx (neg)
*pt9*	72	M	Larynx
*pt10*	65	M	Rhinopharynx
*pt11*	51	M	Larynx (neg)
*pt12*	70	M	Larynx
*pt13*	53	M	Rhinopharynx
*pt14*	43	M	Oropharynx
*pt15*	68	M	Larynx
*pt16*	68	M	Larynx
*pt17*	83	M	Tongue
*pt18*	68	M	Skull base
*pt19*	86	F	Thyroid
*pt20*	33	M	Laterocervical
*pt21*	70	M	Larynx
*pt22*	58	F	Larynx
*pt23*	43	M	Larynx (neg)

**Table 2:** Scoring system used to evaluate the registration quality of PET with MR images.

Score	Meaning
0	Case unusable
1	Alignment of major anatomical structures is sufficient but localization of tumors is not perfectly corresponding
2	Alignment of major anatomical structures is good and localization of tumors corresponds in PET and MRI

**Table 3:** Evaluation results: registration accuracy scores expressed as mean ± standard deviation.

	PETMRo	PETMRreg_ELX_^RR^	PETMRreg_ELX_^DR^	PETMRreg_MRD_^RR^	PETMRreg_MRD_^DR^
Qualitative score	2.0 ± 0.0	0.70 ± 0.84	1.35 ± 0.70	0.76 ± 0.92	0.89 ± 0.80
Dice score	0.82 ± 0.07	0.36 ± 0.23	0.45 ± 0.24	0.37 ± 0.22	0.41 ± 0.23
